# Population prevalence of ultrasound features of osteoarthritis in the hand, knee and hip at age 63 years: the Newcastle thousand families birth cohort

**DOI:** 10.1186/1471-2474-15-162

**Published:** 2014-05-19

**Authors:** Ajay M Abraham, Mark S Pearce, Kay D Mann, Roger M Francis, Fraser Birrell

**Affiliations:** 1 Northumbria Healthcare NHS Trust, Northumberland, UK; 2 Institute of Health & Society, Newcastle University, Newcastle upon Tyne NE1 4LP, UK; 3 Newcastle upon Tyne Hospitals NHS Foundation Trust, Newcastle upon Tyne, UK; 4 Institute for Ageing and Health, Newcastle University, Newcastle upon Tyne, UK

**Keywords:** Osteoarthritis, Ultrasonography, Prevalence, Epidemiology

## Abstract

**Background:**

Musculoskeletal ultrasound has been found to be more sensitive than radiographs in detecting osteophytes. Our objective was to measure the prevalence of features of osteoarthritis (OA), in the dominant hand, knees and hips using ultrasound, within the Newcastle Thousand Families birth cohort.

**Methods:**

Participants were aged 61–63 (mean 63) years. Knee images were scored for presence of osteophytes and effusion. Hip images were scored for the presence of osteophytes and femoral head abnormality. The first carpometacarpal joint, metacarpophalangeal, proximal interphalangeal and distal interphalangeal joints of the index finger (dominant hand) were imaged for osteophytes.

**Results:**

Among 311 participants, prevalence of osteophytes at the distal interphalangeal joint was 70% while it was 23%, 10% and 41% for index proximal interphalangeal and metacarpophalangeal and thumb base carpometacarpal joints respectively. Prevalence of knee osteophytes was 30%, hip OA was 41%. Prevalence of knee effusions was 24% (right) and 20% (left). Ultrasound evidence of generalised OA (48%) and isolated hand OA (31%) was common, compared to isolated hip or knee OA (5%) and both hip and knee OA (3%).

**Conclusion:**

This is the first study to assess prevalence of ultrasound features of OA in a population-based sample. The higher prevalence of hand/hip OA, when compared to previous radiographic studies, supports the hypothesis that ultrasound is more sensitive than radiography in detecting OA, particularly for osteophytes.

## Background

Osteoarthritis (OA) is the most common form of arthritis and has been reported to affect around 12% of the adult population in both the United States [1] and the United Kingdom [2]. This high prevalence is associated with significant economic [3] and social [4] burden, which is expected to increase with an ageing population and increasing obesity [5].

The definition of OA for epidemiological studies and estimates of prevalence have been areas of established interest. Previous community studies have used radiographs as the main imaging modality to define OA at the hand [6-10], knee [11-15] and hip [10,15-17], despite recognised limitations [18]. Radiographs have shown poor correlation with clinical symptoms [19,20] and might not be adequately sensitive as an outcome measure in clinical trials [21]. Ultrasound is an imaging modality that has been gaining increasing popularity in the assessment of OA [22-24]. The advantages of ultrasound are well described; most importantly it is relatively low cost, non-invasive and lacks exposure to ionising radiation. Ultrasound can identify soft tissue structures such as the synovium; important because synovial inflammation is known to be a predictor of progression of knee OA [25]. The multi-planar ability of ultrasound adds dimensions to imaging that are not possible with radiographs and may lead to easier recognition of osteophytes than when compared to radiographs [26]. The use of ultrasound might also help to explain the reported discordance between clinical symptoms and imaging in OA. We have previously demonstrated the reliability and validity of ultrasound in detecting features of knee OA in community participants [27]. Thus, ultrasound imaging has the potential to become an extremely useful tool in studying features of OA in the community.

The hypothesis of this study was that the use of a more sensitive imaging modality (ultrasound) would lead to a different (most likely higher) prevalence estimate for OA at the dominant hand, knees and hips when compared to previous radiographic studies. Osteophytes were imaged at the hand, knees and hips; femoral head abnormality at the hips and synovial effusion at the knees, since these are ultrasound features of OA that have a definite cut-off to define presence or absence. The Newcastle Thousand Families birth cohort presented an ideal setting of unselected individuals from the community to perform this study.

## Methods

### Participants

The Newcastle Thousand Families Study began as a prospective study of all 1142 children born in May and June 1947 to mothers resident in Newcastle upon Tyne, a city in Northern England [28]. The health, growth and development of the cohort were followed in detail up to age 15 years and the cohort also underwent a major follow-up at age 49–51 years. Between October 2009 and March 2011, health and lifestyle questionnaires were sent out for completion and return and study members were invited to attend for a clinical assessment which included ultrasound examination of their dominant hand, both knees and both hips, as well as anthropometric measures such as body mass index (BMI), which took place over the same time period. The study received a favourable ethical opinion from Sunderland Local Research Ethics Committee and all study members gave their written consent.

### Ultrasound assessments

Ultrasound assessments were done using the Mylab 70 XVG machine (ESAOTE, Genoa, Italy) by a trained musculoskeletal ultrasonographer (AA). The dominant hand and the knees were imaged using a 10-18 MHz linear transducer, while the hip was imaged using a 6–10 MHz linear transducer with a 9 cm footprint.

#### Knee

Ultrasound of the knees was based on a protocol derived from European League Against Rheumatology (EULAR) guidelines [29] while the ‘Outcome Measures in Rheumatoid Arthritis Clinical Trials (OMERACT) guidelines for synovial effusion [30] were also met. The presence or absence of osteophytes was assessed at the tibial and femoral sites in both knees, with 30° of knee flexion. 30 degrees flexion of the knees was standardised by using the same wedge for all ultrasound assessments. While there are no comparison studies to assess the validity of using 30 degrees of knee flexion (against other angles), the EULAR guidelines [29] and recent inter-rater reliability studies for knee OA [31] affirm the reliability of this angle for assessment of knee osteophytes and effusion. Femoral and tibial osteophytes were assessed in the medial and lateral compartments using medial and lateral longitudinal scan positions, respectively. The probe was placed in a longitudinal position anteriorly on the lateral border of the patella and then moved posteriorly in a dynamic manner to the level of the biceps femoris to assess for lateral osteophytes both at the femur and the tibia. Similarly, the probe was moved longitudinally from the medial border of the patella on the anterior aspect to the semitendinosus posteriorly to assess for medial osteophytes at the femur and tibia. Osteophytes were defined as cortical protrusions at the joint margin seen in two planes [24]. A recent study has produced a novel atlas in an attempt to quantify the grade of osteophyte in a semi-quantitative manner in patients with hand OA [32]. However, at the time of the Newcastle Thousand Families assessments, there was no specific size cut-off to define a knee osteophyte, which could be considered a limitation of this study. Nevertheless, there are no community data to date to validate the use of a size cut-off for the prevalence of osteophytes.

Prevalent knee OA was defined as the presence of at least one osteophyte in the knee joint. Knees that were replaced were not scanned but marked as having prevalent knee OA.

Synovial effusion was defined as an abnormal anechoic or hypoechoic area in the joint that is displaceable and compressible and lacks Doppler signal; as per the OMERACT guidelines [30]. The presence of effusions was identified in the longitudinal supra-patellar position, with the knee in 30° of flexion. A multi-planar approach was used to identify the effusion in the longitudinal view with the probe being swept from the lateral to the medial recess in a dynamic fashion across the supra-patellar pouch. Multiple readings were then taken to estimate the maximum diameter of the effusion. Knee effusion was defined (on a dichotomous scale) as being present if the size was ≥4 mm, as this definition has previously demonstrated significant association with knee pain [33] and significant correlation with advanced radiographic knee OA in a multi centre European study of knee OA [34].

It was not possible to estimate OA prevalence using cartilage thickness measurements at the knee as there is no recommended cut-off to determine presence or absence of OA for the femoral cartilage thickness measured by ultrasound at the knee.

#### Hand

Generous amounts of ultrasonographic gel were used, in addition to a gel pad, to achieve stand-off, for all assessments of the hand joints. This allowed accurate identification of the target structures with minimal image artefacts. Due to practical limitations with time spent by each participant at the research facility, only four joints of the dominant hand were imaged: first carpometacarpal joint (CMC) and the index metacarpophalangeal (MCP), proximal interphalangeal (PIP) and index distal interphalangeal (DIP) joints. The MCP, PIP and DIP joints of the index finger and CMC joint of the thumb were imaged using a dynamic approach with the probe in a longitudinal position and being swept across the whole of the joint for DIP and PIP joints from the anterior to posterior aspect; and across accessible areas for the MCP and CMC joints. The hand joints were placed in a neutral position for all examinations. The pathology that was identified in each hand joint was osteophytes; defined as cortical protrusions seen in two planes [35]. Prevalent OA in each hand joint represented the presence of at least one osteophyte in the individual joint while prevalent hand OA was defined as OA in at least one hand joint.

#### Hip

The hip was scanned in the anterior longitudinal plane (parallel to the femoral neck) with the hip in mild external rotation (for patient comfort) and the knee in 30° of flexion. The pathology identified was presence of osteophytes and femoral head abnormality, as described by Qvistgaard et al. [36]. The probe was placed in the anterior longitudinal plane and moved from a medial to lateral position in a dynamic fashion till the optimum image was identified. Osteophytes were defined as a definite irregularity in the bone cortex of the femoral head or neck, while femoral head abnormality was present if there was flattening or loss of contour of the femoral head. Hips were classified as having prevalent OA if there was presence of either osteophytes or femoral head abnormality. Hips that were replaced due to OA were not scanned, but marked as having prevalent hip OA.

For the purpose of this study, generalised OA was said to be present if the participant had ultrasound features of OA in at least two out of the three joint groups (hand, knee and hip) that were studied.

### Statistical analyses

Continuous variables were compared between groups using t-tests, while associations between categorical variables were assessed using chi-squared tests. Inter-rater reliability for ultrasound scoring was performed by analysing the scores of a second trained musculoskeletal ultrasonographer, who scored the images for the same pathologies in all joint sites among 25 participants. Kappa statistics were used to measure the level of agreement between the two observers. The statistical software package, Stata, version 10 (Statacorp, College Station, TX) was used for all analyses.

## Results

Of the 349 individuals who attended the clinical assessments, 311 were scanned, 55% of which were women. Mean age was 63 years. Mean BMI was 26.5 (SD 4.2) kg.m^-2^ and 61% of participants had a BMI ≥ 25 kg.m^-2^. There was a significantly higher proportion of women at this assessment of the cohort when compared to the original birth cohort (p = 0.008).

### Reliability of ultrasound assessments

The Kappa inter-rater reliability for osteophytes or effusion at the knee was moderate to excellent, with values ranging from 0.49 to 0.92 (Table 1). Agreement at the hand was moderate to substantial (0.50, 0.69), while at the hip it was moderate.

**Table 1:** Inter-rater reliability of ultrasound features of OA at knee, hand and hip joints

	**Kappa (95% CI)**
Right medial femoral knee osteophyte	0.86 (0.72, 0.99)
Right lateral femoral knee osteophyte	0.92 (0.82, 1.00)
Left medial femoral knee osteophyte	0.77 (0.60, 0.93)
Left lateral femoral knee osteophyte	0.79 (0.62, 0.95)
Right medial tibial knee osteophyte	0.75 (0.54, 0.96)
Right lateral tibial knee osteophyte	0.49 (0.18, 0.81)
Left medial tibial knee osteophyte	0.73 (0.53, 0.93)
Left lateral tibial knee osteophyte	0.65 (0.42, 0.88)
Right knee effusion	0.92 (0.82, 1.00)
Left knee effusion	0.79 (0.61, 0.96)
CMC OA	0.69 (0.42, 0.95)
MCP OA	0.50 (0.02, 0.99)
PIP OA	0.62 (0.30, 0.93)
DIP OA	0.69 (0.39, 1.00)
Right hip OA	0.69 (0.36, 1.00)
Left hip OA	0.40 (0.07, 0.74)
Any hip OA	0.47 (0.19, 0.75)

### Prevalence of OA at the three joint sites

**Figure 1:**
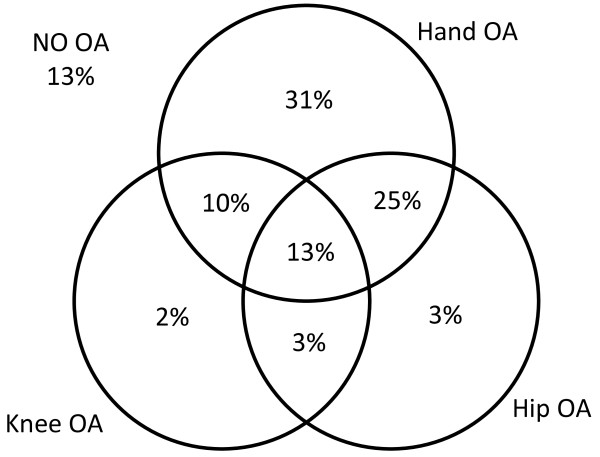
Venn diagram detailing pattern of OA (%).

Prevalence of osteophytes in the dominant hand was high at the DIP joint at 70%, while it was 23%, 10% and 41% for index PIP, index MCP and thumb base CMC joints, respectively (Table 2). Hand OA prevalence was higher among females compared to males (p = 0.005). Prevalence of knee osteophytes was 22%, 25% and 30% for right, left and “any” knee, respectively. There was no significant difference of knee osteophyte prevalence between males and females (p = 0.8). The prevalence of knee effusions was 24% and 20% in right and left knees, respectively; with males showing a non-significant trend to higher prevalence (p = 0.1). The prevalence of hip OA was higher than described in radiographic surveys, with 26%, 30% and 41% in right, left and “any” hip, respectively. Males had higher prevalence of hip OA (p = 0.02). Ultrasound evidence of generalised OA (48%) and isolated hand OA (31%) were common, compared to isolated hip or knee OA (5%) and both hip and knee OA (3%) (Figure 1).

**Table 2:** Prevalence of ultrasound features of OA in the knee, hip and hand joints

	**Total: n = 311**	**Male: n = 137**	**Female: n = 172**
	**Proportion (95% CI)**	**Proportion (95% CI)**	**Proportion (95% CI)**
OA any knee	30% (0.25, 0.35)	28% (0.20, 0.36)	32% (0.25, 0.39)
OA right knee	22% (0.17, 0.27)	20% (0.13, 0.26)	24% (0.17, 0.30)
OA left knee	25% (0.20, 0.30)	23% (0.16, 0.30)	27% (0.20, 0.33)
OA any hip	41% (0.35, 0.46)	44% (0.36, 0.53)	38% (0.30, 0.45)
OA right hip	26% (0.21, 0.31)	26% (0.19, 0.34)	26% (0.19, 0.33)
OA left hip	30% (0.25, 0.35)	34% (0.26, 0.42)	26% (0.19, 0.33)
OA hand	78% (0.73, 0.82)	67% (0.60, 0.75)	86% (0.81, 0.91)
OA CMC	41% (0.35, 0.46)	33% (0.25, 0.40)	47% (0.40, 0.55)
OA MCP	10% (0.07, 0.13)	9% (0.04, 0.13)	11% (0.06, 0.16)
OA PIP	23% (0.18, 0.28)	21% (0.14, 0.28)	25% (0.18, 0.31)
OA DIP	70% (0.65, 0.75)	56% (0.48, 0.64)	81% (0.75, 0.86)

## Discussion

The estimated prevalence of features of OA in this cohort was 78% for the dominant hand, 30% for knee and 41% for hip. We have, demonstrated a higher prevalence of OA using ultrasound compared to previous studies using radiographs, especially in the hand and the hip joints.

For knee OA, the prevalence estimates of this study are not dissimilar to that found in the 55–64 year age group of the Johnston County Osteoarthritis project [12], which had a high proportion of overweight (74% had a BMI ≥ 25 kg.m^-2^; compared to 61% in our cohort). The prevalence estimate of 33% radiographic knee OA in the Framingham study [11] is also similar to our study. However, the Framingham cohort had participants with a mean age of 73 years; which is ten years older than the Newcastle Thousand Families cohort currently. It is possible that the healthier lifestyles that may have been prevalent at the time of the Framingham assessment (~30 years ago) would have brought the prevalence estimate of OA down in that cohort. A study of 1729 community dwelling individuals from Nottingham, UK, with a mean age of 64 years (mean BMI 26.8) estimated the prevalence of symptomatic radiographic knee OA to be much lower at 11%, despite the cohort including participants from a previous knee study on pain [10]. The differences in the above estimates could also be explained by the fact that the above three studies used different criteria to define radiographic knee OA. The Nottingham study required the presence of knee pain with a grade 2 osteophyte, while the Framingham and Johnston County studies required a Kellgren-Lawrence (K-L) grade of ≥ 2. The lower prevalence of OA in the Nottingham cohort could therefore be explained by the need for symptoms (knee pain) in addition to radiographic features of OA.

A further report from The Johnston County Osteoarthritis Project study of 2637 community dwelling men and women aged 45 years and older showed an overall prevalence of radiographic hip OA of 27.6% [16]. The prevalence increased with age; the 55–64 year group had a prevalence of 23% and this increased to 31% in the 65–74 year age group. These estimates were higher than the previously published NHANES 1 study [37]. The authors of the Johnston County study suggested that this might have been due to a higher prevalence among rural (vs urban) participants in Johnston County. Other reasons include the possibility of changes in hip OA risk factor profiles over time as well as variation in radiographic techniques and interpretation. While the definition of radiographic hip OA in the Johnston County study and NHANES 1 were the same (K-L grade of ≥ 2), it is still feasible that the images from the NHANES 1 study were under-read. One of the reasons for the high prevalence of hip OA noted in the Newcastle Thousand Families cohort could have been because the ultrasound definition for presence of an osteophyte was more sensitive than the radiographic K-L score of ≥ 2 (which requires a “definite” osteophyte, as opposed to the “doubtful” osteophyte seen with a K-L score of 1).

1467 men and 1519 women of the Medical Research Council’s (MRC) National Survey of Health and Development had a clinical hand examination performed at age 53 [6]. The prevalence of OA (defined by examination, but not by imaging) was 21%, 12% and 8% in any of the DIP, PIP and CMC joints, respectively, among women. In men, the corresponding values were 14%, 8% and 4%. However, the participants were 10 years younger than the Newcastle Thousand Families cohort were when examined and were therefore likely to have lower prevalence estimates. Another reason for the low estimates in the MRC survey is that ultrasound is likely to be more sensitive than clinical examination in the detection of osteophytes. A cohort of 489 participants from a family study of nodal OA in Nottingham in 2004, had a prevalence estimate for radiographic OA of 46% in the right index finger and 73.3% for overall hand OA [10], results quite similar to our study. The mean age of the Nottingham cohort was 65.7 years with a high proportion of women (82.6%). However, this was not a random population sample as the Nottingham participants were selected for their higher risk of prevalent hand OA. Factors known to influence the prevalence of hand OA such as occupation [38], grip strength [39] and other systemic factors such as use of oestrogen [40] and obesity [41] might be responsible for some of the differences observed.

In contrast to the Nottingham radiographic cohort study [10], we did not find any significant differences between the right and left knee for osteophyte detection using ultrasound. Similar to their findings though, there was no significant difference between the right and left hip osteophyte prevalence. Our findings are, however, in line with the Zoetermeer Survey that showed no evidence of any right-left differences of radiographic OA at the knee and hip joints [14]. This suggests that bio-mechanical stresses act equally on both sides of the lower limb joints. Equally, it might also suggest that genetic and systemic factors such as oestrogen [40] and obesity [41] might play a larger role in the pathophysiology of OA at the knee and hip, than bio-mechanical stress. We report that the prevalence of isolated knee and/or hip OA was extremely low in this cohort (8%); while isolated hand OA was particularly high (31%). This high prevalence of hand OA suggests that ultrasound defined hand osteophytes may be a predictor of a more generalised form of OA affecting the knee and the hip. Cooper et al. [42] demonstrated that there is no single threshold number of joint sites that could be used to define generalised OA. However, for the purpose of this study, we used a definition that would include two out of three joint sites (hand, knee and hip). However, a longitudinal follow-up of these participants will be required to consolidate this hypothesis, since it is possible that biases such as a survival bias might have influenced the cross sectional results noted above.

We did not find a higher prevalence of knee OA in women, as has been noted in previous studies, such as the Framingham study [43]. However, the Framingham study showed an increasing prevalence of OA with age, particularly in women. The Framingham participants were aged 63 years and older (and hence older than the Newcastle Thousand Families Cohort) and the higher prevalence of knee OA in women in Framingham was seen mostly in the older age groups. The prevalence of knee OA in the 63–69 year age group was higher in men (30%) when compared to women (25%) but in the ≥80 year age group, there was a higher prevalence seen in women (53%) when compared to men (33%). This might explain why the prevalence of knee OA was not higher in females in the Newcastle Thousand Families Study, as the participants were aged only 63 years and it is likely that at this (relatively young) age there is no significant difference in knee OA prevalence between the sexes. Indeed, a meta-analysis study of sex differences in knee OA demonstrated that among those aged ≥55 years of age, there was a significantly lower pooled risk of prevalent radiographic knee OA, but this significance was lost when looking at those <55 years of age [44].

The prevalence of knee effusions was remarkably high in this study (just under a quarter of knees had ≥4 mm knee effusion on ultrasound), considering the subjects were a population sample, and not selected for symptoms. The high prevalence could potentially be explained by defining effusion with a low cut-off (i.e. 4 mm). However, this definition has been validated against symptoms in a multi-centre European study [34]. It was also interesting to note that males had a trend towards a higher prevalence of knee effusion than females, although this did not quite reach statistical significance (p = 0.1). Since there is increasing evidence that inflammation predicts knee OA progression [25,45], this would suggest that males in this cohort might be at a higher risk of rapidly progressive OA. Further longitudinal follow up of the cohort should help to address the issue of predictive validity of knee effusion identified by ultrasound.

A strength of our study is that it uses a well characterised population-based cohort to estimate the prevalence of features of OA at different joint sites using a sensitive imaging modality, namely ultrasound. This is the first study to estimate prevalence of certain ultrasound features of OA in the community. Although there were a few variations in inter-rater agreement across the joints, the level of agreement found on inter-rater reliability of ultrasound images in this study helps to reduce some of the concern about the subjective nature of ultrasound scoring of images.

There were a few limitations to this study. We mainly imaged the bony parameters in the joints and did not include prevalence estimates for cartilage thickness. This is because of the lack of a clear cut-off in the knee to define “cartilage thinning” as well as the limited ability of ultrasound to identify cartilage morphology in the hand joints due to technical feasibility. However, this would suggest that the prevalence estimates of knee OA in our study are likely to be under estimates. The study populations, definitions and image acquisitions in previous radiographic studies of OA have varied considerably [46] and hence any direct comparisons with this study are difficult to make. Another limitation is that radiographic osteophytes were not reported and hence comparison of radiographs with ultrasound imaging was not done. Power doppler synovitis was not assessed in this study, as the knee and hip joints are considered too deep for accurate assessment of power doppler signal and power doppler in hand OA has not yet been shown to demonstrate adequate construct validity. Kappa statistics for inter-rater reliability of ultrasound imaging were only calculated on a small proportion of the participants. This might explain the uncertain estimates and large confidence intervals noted at a few of the joint sites. Furthermore, the inter rater reliability at the hip was moderate in this study, rather than the substantial or excellent reliability at other sites. Further work to standardise the acquisition techniques and reading of hip ultrasound images for features of OA will help to decrease imprecision of prevalence estimates due to the imaging modality itself. Also, this study was performed among members of a birth cohort born in the city of Newcastle upon Tyne, UK, which might reduce the external validity of the findings. Another drawback is that inferences can only be made for this particular age group of subjects (aged 61–63 years). However, this is an age group that has particular public health importance, as the prevalence of OA in this age group is quite high and effective risk factor modification (e.g. diet and exercise) is still likely to be feasible.

## Conclusion

This is the first study to assess the prevalence of ultrasound features of OA in the community. The higher prevalence of OA in the hand and hip in this study, when compared to previous radiographic studies, supports the hypothesis that ultrasound is more sensitive than radiography in detecting the structural changes of OA, particularly for osteophytes, although imaging only the dominant hand determines that this might be an underestimate. Direct comparisons with radiographs in community studies will help to further confirm this hypothesis. Follow up of this and other cohorts could establish the value of ultrasound in predicting radiographic change and test the hypothesis that isolated hand OA on ultrasound predicts the development of generalised OA.

## Abbreviations

95% CI: 95% confidence interval; BMI: Body mass index; DIP: Index distal interphalangeal joint; EULAR: European league against rheumatology; CMC: First carpometacarpal joint; MCP: Index metacarpophalangeal joint; MRC: Medical research council; OA: Osteoarthritis; OMERACT: Outcome measures in rheumatoid arthritis clinical trials; PIP: Proximal interphalangeal joint.

## Competing interests

The authors declared that they have no competing interests.

## Authors’ contributions

MSP, FB and RMF conceived and designed the study, and obtained the funding. AMA carried out the ultrasound assessments and MSP was responsible for directing both the overall clinical assessment and the data collected by questionnaire (the aspects included in this paper were designed by MSP, AMA, FB and RMF). AMA and KDM did the statistical analysis, supervised by MSP. AMA and MSP drafted the paper with critical contributions from all other authors. All authors read and approved the final manuscript.

## Pre-publication history

The pre-publication history for this paper can be accessed here:

http://www.biomedcentral.com/1471-2474/15/162/prepub
